# Identifying iNOS and glycogen as biomarkers for degenerated cerebellar purkinje cells in autism spectrum disorder: Protective effects of erythropoietin and zinc sulfate

**DOI:** 10.1371/journal.pone.0317695

**Published:** 2025-02-13

**Authors:** Abdulaziz M. Al-Garni, Sara A. Hosny, Faris Almasabi, Ayed A. Shati, Norah M. Alzamil, Asmaa M. ShamsEldeen, Asmaa A. El-Shafei, Fahaid Al-Hashem, Hind Zafrah, Amro Maarouf, Bahjat Al-Ani, Ismaeel Bin-Jaliah, Samaa S. Kamar

**Affiliations:** 1 Psychiatry section, Department of Medicine, College of Medicine, King Khalid University, Abha, Saudi Arabia; 2 Department of Psychiatry, School of Medicine, Queen’s University, Kingston, Ontario, Canada; 3 Medical Histology Department, Faculty of Medicine, Cairo University, Cairo, Egypt; 4 Department of Physiology, College of Medicine, King Khalid University, Abha, Saudi Arabia; 5 Department of Child Health, College of Medicine, King Khalid University, Abha, Saudi Arabia; 6 Department of Family and Community Medicine, College of Medicine, Princess Nourah bint Abdulrahman University, Riyadh, Saudi Arabia; 7 Physiology Department, Faculty of Medicine, Cairo University, Cairo, Egypt; 8 Department of Clinical Biochemistry, Russells Hall Hospital, Dudley, United Kingdom; University of Nebraska Medical Center College of Medicine, UNITED STATES OF AMERICA

## Abstract

Autism spectrum disorder (ASD) is a collective neurodevelopmental disorder affecting young children and accounting for 1% of the world’s population. The cerebellum is the major part of the human brain affected by ASD and is associated with a substantial reduction in the number of Purkinje cells. An association between ASD and the expression of the nitrosative stress biomarker inducible nitric oxide synthase (iNOS), as well as glycogen deposition in damaged Purkinje cells, has not been previously reported in the medical literature. To explore this correlation, young rats were injected with propionic acid (PPA) (500 mg/kg) for 5 days (model group), while the protection groups were treated with either erythropoietin (EPO, 5,000 U/kg) or 2 mg/kg zinc sulfate immediately after the PPA injections. ASD-like features were developed in the model group, as evidenced by cerebellum damage (degeneration of Purkinje cells) and cerebellar dysfunction (behavioral impairment). This study documented the *exclusive* expression of iNOS in the degenerated Purkinje cells, along with glycogen deposition in these cells. Additionally, PPA significantly (p < 0.001) modulated cerebellar tissue levels of mammalian target of rapamycin (mTOR), gamma-aminobutyric acid (GABA), GABAA receptor, serotonin, the marker of neuronal loss (calbindin D28K), and social interaction deficit. Some of these parameters were differentially protected by EPO and zinc sulfate, with the former providing greater protection than zinc sulfate. Furthermore, a significant correlation between the iNOS score and these parameters associated with ASD was observed. These findings demonstrate the colocalization of iNOS and glycogen in the damaged Purkinje cells induced by ASD, along with the modulation of ASD parameters, which were protected by EPO and zinc sulfate treatments. Thus, these potential novel biomarkers may offer possible therapeutic targets for the treatment of ASD.

## Introduction

Autism spectrum disorder (ASD) (also known as autism) is a neurodevelopmental disorder that tends to run in families and is described by impaired communication and social interaction, repetitive and abnormal movements, sensory defects, and infrequently, self-injury [1]. An estimated 1 in 59 children in the United States have ASD by the age of 8 years (a prevalence rate of 1.7%) with a median diagnosis age of 52 months [2]. ASD has a male preponderance, with an estimated ratio of 4:1 [3]. The hippocampus and amygdala have been implicated as the major part of the brain affected by ASD [4,5]. There has also been interest in the cerebellum, with ASD-like phenotype being associated with an extensive decrease in the number of Purkinje cells [6]. Post-mortem examination of brain tissue from ASD patients demonstrate decreases in the cerebellum’s gray and white matter, as well as a reduction in the average number and size of Purkinje cells and the cerebellum’s output neuron, indicating a significant role in ASD pathophysiology [7].

The short-chain fatty acid propionic acid (PPA) is an endogenously synthesized carboxylic acid produced by gut microorganisms and commonly used as a food preservative. An association between gastrointestinal diseases and ASD has been reported [8]. PPA also passes the blood-brain barrier, where it is taken up by neuronal cells within the brain, causing intracellular acidification. The latter is thought to affect neurotransmitter release, neuronal transmission and behavior [8]. PPA has been linked to various neuro-inflammatory and behavioral changes in ASD-like rat models where different PPA methods of administration (subcutaneous, intraperitoneal, intra-gastric lavage, and intra-cerebroventricular routes) have been applied [9]. This makes the *endogenous* neurotoxic compound PPA, an ideal candidate in mimicking ASD in animal models, over *exogenous* neurotoxic foreign compounds.

The glycoprotein hormone erythropoietin (EPO) is produced by the peritubular interstitial cells of the kidney in response to cellular hypoxia or anaemia. It plays an important role in the production of red blood cells (erythropoiesis) by the bone marrow [10]. However, EPO’s physiological role is not just limited to erythropoiesis but also includes vasoconstriction [11], the formation of new blood vessels (angiogenesis) [12], promotion of cell survival [13], and protection against many brain diseases [14]. Within the brain, the receptors for EPO are detected in the cerebral cortex, midbrain, hippocampus, and internal capsule [15]. Several clinical and non-clinical research studies have demonstrated EPO’s neuroprotective and neurotrophic efficacy in various neuropsychiatric disorders, including neuropathies, epilepsy, multiple sclerosis, Alzheimer’s disease, and traumatic brain injury [16]. In animal models of autism induced by (i) bacterial toxin lipopolysaccharides (100 μg/kg) that were injected to pregnant rats thereby producing ASD-like symptoms in the littermates; and (ii) PPA (500 mg/kg) injected to 3 week-old rats, EPO showed modulatory effects on neurogenesis as well as anti-inflammatory, antioxidant and anti-excitotoxic properties, in addition to improved memory and learning performance, and protection against ASD development [17,18].

Zinc is required for normal brain development. It serves as a cofactor for DNA and RNA polymerases, as well as histone catalases and DNA ligase. Consequently, the majority of processes in protein synthesis and gene expression in the CNS involve zinc [19]. Zinc is primarily found pre-synaptically, and more frequently in glutamatergic neurons. Post-synaptic receptors such as N-Methyl-D-aspartate, GABA, and voltage-gated calcium channel receptors have been demonstrated to be modulated by zinc release [20]. A significant association between zinc deficiency and autism was reported in a retrospective controlled trial study assessing serum levels of zinc in 72 patients with ASD versus 234 non-ASD persons [21]. This study demonstrated that 86% of the screened patients had zinc deficiency compared to 24% in the control non-ASD group, after adjusting for age and sex as well as chromium and manganese blood levels [21].

mTOR, reactive oxygen species (ROS), and reactive nitrogen species (RNS) are known inducers of neuroinflammation and neurodegenerative pathologies and are associated with the development of ASD via different cell signaling pathways [22–25]. Targeting mTOR, was therefore suggested as a potential strategy for the treatment of ASD [26]. Furthermore, glycogen dysregulation in degenerated Purkinje cells and other brain areas are reported in patients and mice with mutations associated with neuronal loss and epilepsy [27,28]. However, the association between ASD and cerebellar iNOS, glycogen dysregulation, and mTOR activation in human and animal models has not been previously investigated. Thus, the aim of this study was to investigate whether such an association exists and whether EPO and zinc sulfate treatments could differentially inhibit these potential deleterious markers. The use of EPO and zinc sulfate to protect against PPA-induced ASD in this study is supported by the aforementioned reports in addition to a lack of effective medication available for the treatment of ASD.

## Materials and methods

### Animals

Young male albino rats, with an average age of 3 weeks and weighing about 100 g, were housed in a clean facility with a well-ventilated clean room at stable temperature. The rats had free access to food and water throughout the experiment. The experimental protocol followed the animal research guidelines approved by the Institutional Animal Care and Use Committee of Cairo University (IACUC No. CU/III/F/48/22).

### Experimental design

The rats were equally separated into 4 groups (n = 8) as follows: The control group (Control), consisting of rats injected subcutaneously (SC) with the vehicle. The model group (PPA) comprised rats given 500 mg/kg of PPA (Sigma-Aldrich, St. Louis, Missouri, USA) dissolved in 0.1 M PBS (250 mg/mL, pH 7.4) administered SC once a day for 5 successive days [9]. The treated groups were as follows: (i) PPA+ZnSO4, rats received PPA at the same dose and by the same route as in the model group, followed by SC injection of ZnSO4 (2 mg/kg, Sigma-Aldrich, St. Louis, Missouri, USA) once daily for 2 weeks [29]. (ii) PPA+EPO, rats received PPA as mentioned above, followed by intraperitoneal injection of recombinant human EPO (Santa Farma, Istanbul, Turkey) at a dose of 5,000 U/kg once daily for 2 weeks [30]. All rats underwent neurological assessments at the end of the investigation using the elevated plus maze and social interaction tests to assess the progression of autistic features. Then, the rats were euthanized immediately after the end of the neurological assessments by intraperitoneal injection of ketamine-xylazine (100 mg/kg) anesthesia, followed by transcardial perfusion with 10% formalin. The cerebellum harvested from each rat was divided into two hemispheres for histological and biochemical analysis.

### Neurological examination

Assessment of anxiety-like behavior and social interaction and engagement were conducted using the elevated plus maze test and a white apparatus (50 × 40 × 40 cm box) respectively, as recently described by our group [18]. A video camera recorded the rats’ behaviors, including open arm entry, the time spent in the open arm, and the number of head dips. Additionally, social interaction time between rats was recorded over 20 minutes.

### Measurements of cerebellar GABA and serotonin

As recently described by our group [18], tissue homogenates were prepared from the harvested right cerebellar hemispheres and used to measure tissue levels of GABA and serotonin. ELISA immunoassay kits were used to measure GABA (ALPCO Diagnostics, Salem, NH, USA) and serotonin (Immuno-Biological Laboratories, IBL, Hamburg, Germany).

### Histological studies

The left cerebellar hemispheres were fixed in 10% formalin for 48 hours, then processed for paraffin blocks. 5 μm thickness sections were cut and subjected to hematoxylin and eosin stain (H&E) and Periodic Acid Schiff (PAS) to illustrate the morphological changes and polysaccharides content.

### Immunohistochemistry of iNOS and calbindin D28K

Immunohistochemistry of cerebellum specimens for iNOS and Calbindin D28K was carried out as we recently reported [18]. Following the antigen retrieval process, cerebellar tissue sections were incubated with anti- iNOS (Abcam, Cambridge, UK) or anti-calbindin D28K (Santa Cruz Biotechnology, Dallas, TX, USA) antibodies overnight in a humidity chamber. Subsequently, the secondary antibody was added for 30 minutes, and sections were co-stained with Meyer’s hematoxylin.

### Quantitative real-time polymerase chain reaction (qRT-PCR) of GABA_A_ receptor and mTOR

qRT-PCR was used to measure the quantitative expression of the GABA_A_ receptor and mTOR gene expression. Homogenized cerebellar samples of all the studied groups were processed, and total RNA was isolated using TRIzol (Life Technologies, USA). The single-stranded cDNA was created using Thermo Scientific cDNA kit (#K4374966) for RT-PCR. The PCR was conducted using GoTaq Green Master Mix (Promega), PCR grade water, and specific primers for these mentioned genes. The relative expression levels were calculated using the comparative Ct technique with β-actin as a reference (Applied Biosystems, USA).

**Table:** 

Gene	Primer sequence
**GABA**_**A**_ **R**	Forward primer: *5* *′* *- TGCCTGTGTTTCCCTAAAACG-3* *′* Reverse primer: *5* *′* *- AGGCAGGACCAAATCAAACAAT-3* *′*
**mTOR**	Forward primer: *5* *′* *- GGCTTCTGAAGATGCTGTCC-3* *′* Reverse primer: *5* *′* *- GAGTTCGAAGGGCAAGAGTG-3* *′*
**β-actin**	Forward primer: *5* *′* *- GGAGATTACTGCCCTGGCTCCTA-3* *′* Reverse primer: *5* *′* *- GACTCATCGTACTCCTGCTTGCTG-3* *′*

### Morphometric study

An Olympus light microscopy (Japan) was used to examine 10 randomly chosen high-power fields (x400) per section. The images were analyzed with the “Leica Qwin 500C” image analysis system (Leica Imaging System Ltd, Cambridge, UK) to obtain quantitative data derived from the above procedures.

### Statistical analysis

Data were coded and entered using the statistical package for the Social Sciences (SPSS) version 28 (IBM Corp., Armonk, NY, USA). Comparisons between groups were done using analysis of variance (ANOVA) with multiple comparisons post hoc test in normally distributed quantitative variables while non-parametric Kruskal-Wallis test and Mann-Whitney test were used for non-normally distributed quantitative variables. Correlations between quantitative variables were done using Spearman correlation coefficient. P-values less than 0.05 were considered as statistically significant**.**

## Results

### Induction of autism spectrum disorder (ASD) is inhibited by EPO and zinc sulfate treatments

**Fig 1:**
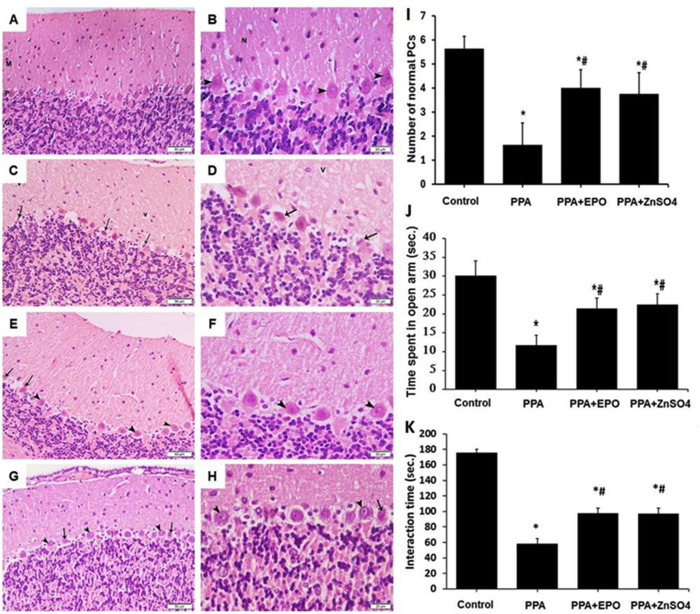
EPO and zinc sulfate protect against PPA-induces ASD in rats. At the end of the experiment, anxiety-like behavior and cerebellar tissue architecture were assessed. (A-H). H&E images (A,C,E,G 200x; B,D,F,H 400x) of cerebellum tissues obtained from the control (A,B), the model (PPA)(C,D), EPO-treated (PPA+EPO) (E,F), and zinc-treated (PPA + ZnSO_4_ (G,H) groups of rats are visualized using light microscopy. The bar graph in (I) represents the quantitative analysis of PC number in cerebellar sections prepared from these rat groups. The bar graphs in (J and K) represent the quantitative analysis of the time spent in open arm (J) and the social interaction time (K) for the groups above. Findings exemplify mean ( ± SD). Presented p values are all significant. * p < 0.05 versus control, #p < 0.05 versus PPA. PPA: propionic acid; ASD: autism spectrum disorder; N: acidophilic neuropil; V: vacuolation in the neuropil of the molecular layer; EPO: erythropoietin; ZnSO_4_: zinc sulfate; PC: Purkinje cell; M: molecular layer; P: Purkinje cell layer; G: granular layer.

Degeneration of Purkinje neurons and anxiety-like behaviour are well-known markers of ASD [31,32]. To test our working hypothesis, we first modelled ASD in rats by assessing these parameters in the model group (PPA) and control untreated rats as well as the treated groups. Using basic histology staining, we assessed the integrity of tissue structural changes of the cerebellar cortex, where Purkinje cells are located. Harvested cerebellum tissues were stained with H&E. Representative images of the cerebellum from the control rats (Fig 1A,B) showed normal cerebellum architecture with unremarkable molecular, Purkinje, and granular layers. The Purkinje layer consisted of one row of large Purkinje cells with acidophilic cytoplasm and single central vesicular nuclei (arrowhead). In contrast, the pathological changes in cerebellum sections from the model group (Fig 1C,D) displayed distorted Purkinje cells (arrow) with smaller sizes, pyknotic nuclei and empty pericellular spaces, along with separations between granule cells in the granular layer. The EPO-treated group (Fig 1E,F) showed largely normal histological architecture of the cerebellar cortex and Purkinje cells, that being of a large regular shape with single central vesicular nuclei (arrowhead), except for a few irregular darkly stained Purkinje cells with pyknotic nuclei (arrow). Treatment with zinc sulfate (Fig 1G,H) revealed some apparently normal Purkinje cells with vesicular central nuclei and prominent nucleoli (arrowhead), while others still appeared distorted with deeply stained pyknotic nuclei (arrow). The molecular and granular layers appeared unaffected. Quantitative analysis of normal Purkinje cell numbers obtained from tissue sections stained with H&E (Fig 1I) demonstrated significant (p < 0.05) protection by EPO and zinc sulfate.

An anxiety test performed two days before the rats were culled demonstrated significant (p < 0.05) differences in the model group compared to the control rats relating to time spent in the open arm (Fig 1J) and social interaction time (Fig 1K), which were significantly (p < 0.05) but partially protected by EPO and zinc sulfate.

### Exclusive iNOS expression in damaged Purkinje cells protected by EPO and zinc sulfate treatments

**Fig 2:**
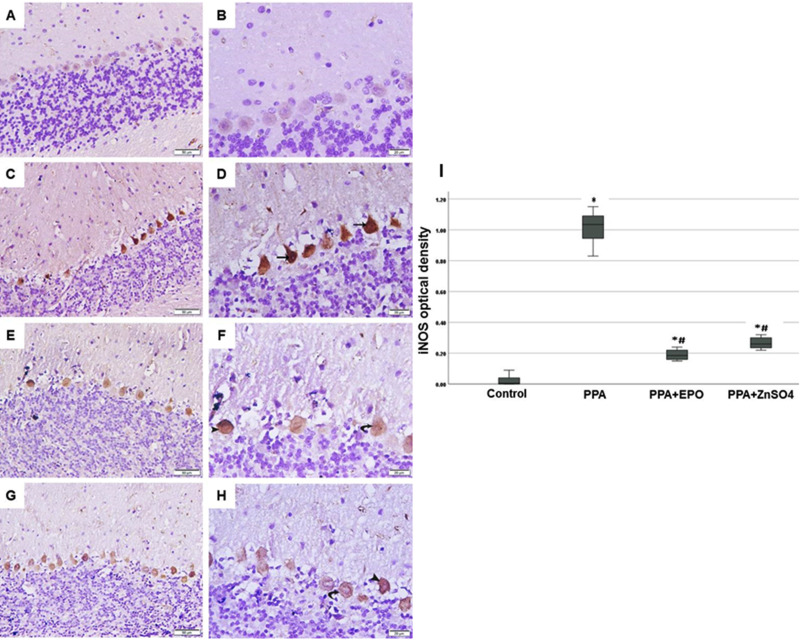
EPO and ZnSO_4_ inhibit PPA-induced iNOS expression in Purkinje cells. iNOS immunohistochemistry of cerebellar tissue sections from rats 17 days after injection with vehicle to the control group (A,200x; B, 400x) and PPA to the model (C,200x; D, 400x) and treated groups; PPA+EPO (E,200x; F, 400x) and PPA + ZnSO**_4_**(G,200x; H, 400x) are illustrated. Note that arrows in D point to the strong positive iNOS cytoplasmic immunostaining in the degenerated Purkinje cells. Whereas, arrow heads and curved arrows in F and H point to the moderate and mild positive immunostaining, respectively. (I) Degree of iNOS immunostaining in cerebellar sections from groups above is illustrated in a bar chart. Findings exemplify median ±  IQ ±  max/min values. Presented p values are all significant; * p < 0.05 versus control, #p < 0.05 versus PPA. iNOS: inducible nitric oxide synthase; PPA: propionic acid; EPO: erythropoietin; ZnSO_4_: zinc sulfate.

Elevated levels of iNOS gene expression were reported in the hippocampus of mice exposed to maternal separation stress that induced autistic-like behaviours [25]. We tested the hypothesis that ASD induced by propionic acid (PPA) is associated with increased iNOS expression in the cerebellum. Immunohistochemical staining of cerebellar tissue samples prepared from the ASD group (PPA) showed a strong positive iNOS protein expression confined only to damaged Purkinje cells (Fig 2C,D,I) compared to negative iNOS expression in the control group (Fig 2A,B,I). EPO (Fig 2E,F,I) and zinc sulfate (Fig 2G,H,I) treatments significantly (p < 0.001) inhibited iNOS protein expression, with EPO having lower values compared to zinc sulfate but did not reach significance (p =  0.135).

### PPA modulates cerebellum tissue levels of mTOR, GABA_A_ receptor, GABA, serotonin, and calbindin D28K protected by EPO and zinc sulfate treatments

**Fig 3:**
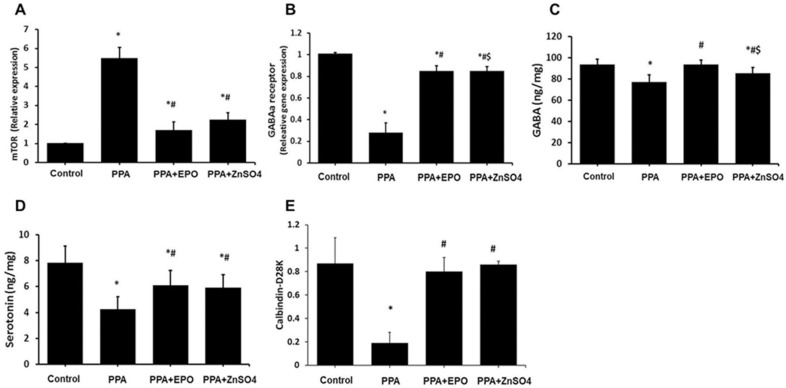
EPO and ZnSO_4_ protect against the dysregulation of cerebellar tissue levels of ASD biomarkers induced by PPA in rats. Tissue levels of mTOR gene expression (A), GABAa receptor gene expression (B), GABA (C), serotonin (D), and calbindin-D28K (E) were assessed in all group of rats at the end of the investigation. Findings exemplify mean ( ± SD). Presented *p* values are all significant. * p < 0.05 versus control, #p < 0.05 versus PPA, $p < 0.05 versus EPO+PPA. ASD: autism spectrum disorder; EPO: erythropoietin; ZnSO_4_: zinc sulfate; PPA: propionic acid; mTOR: mammalian target of rapamycin; GABAa: gamma amino-butyric acid receptor type a; GABA: gamma amino-butyric acid.

Previous studies showed that iNOS is located upstream of mTOR in cell signaling [33], and mTOR inhibition can prevent neuronal death [34]. We next assessed cerebellar levels of mTOR and other ASD biomarkers with and without EPO and zinc sulfate treatments in all rats. In the model group (PPA), there was a significant (p < 0.001) increase in mTOR gene expression levels (Fig 3A), and a significant (p < 0.001) decrease in GABA_A_ receptor gene expression (Fig 3B), GABA (Fig 3C), serotonin (Fig 3D), and calbindin D28K expression in the distorted Purkinje cells and nerve fibers (Fig 3E) compared with the control rats. These ASD-associated changes were considerably mitigated by EPO and zinc sulfate treatments, with EPO showing similar or higher protection than zinc sulfate.

### PPA increases glycogen accumulation in Purkinje cells; protection by EPO and zinc sulfate treatments

**Fig 4:**
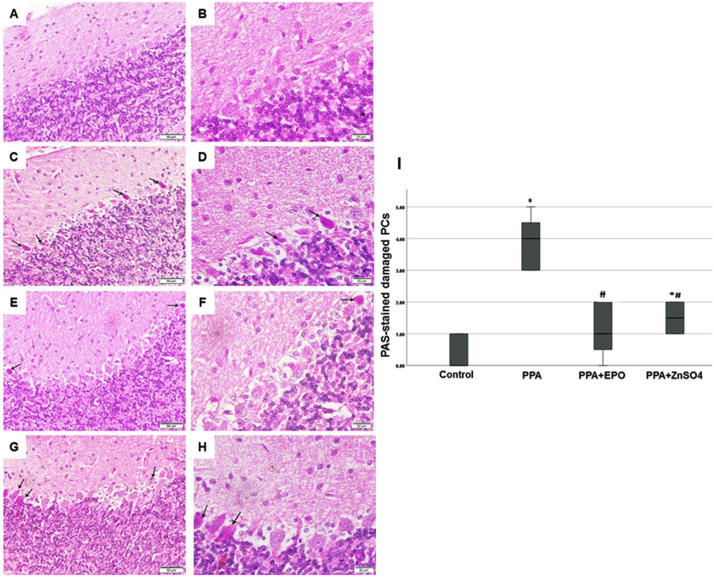
Induction of glycogen accumulation in Purkinje cells by PPA appears to be ameliorated by EPO and ZnSO_4_. PAS stained pictures (A,C,E,G, 200x; B,D,F,H, 400x) of cerebellar tissue sections from rats 17 days after injection with vehicle to the control group (A,B) and PPA to the model (C,D) and treated groups; PPA+EPO (E,F) and PPA + ZnSO_4_ (G,H) are shown. Note that arrows in C and D point to the strong positive PAS stained degenerated Purkinje cells, and arrows in E-H point to few PAS positive cells. (I) Degree of PAS-stained cerebellar tissue sections from groups above are demonstrated in a bar chart. Findings depict median ±  IQ ±  max/min values. Presented p values are all significant; * p < 0.05 versus control, #p < 0.05 versus PPA. PAS: periodic acid Schiff; PPA: propionic acid; EPO: erythropoietin; ZnSO_4_: zinc sulfate.

Forced accumulation of glycogen in Purkinje cells is associated with neuronal loss and locomotion behavioural defects in genetically modified mice [28]. Given the results described above showing behavioural impairment, neuronal loss, and confined expression of nitrosative stress proteins in damaged Purkinje cells, we assessed cerebellar levels of glycogen in our animal model. Tissue sections prepared from all harvested cerebellums were stained with PAS stain, which indicates polysaccharides. Microscopic examination of cerebellum tissue sections from the ASD-like feature group (PPA) showed a strong PAS + ve reaction (arrow) in the degenerated Purkinje cells (Fig 4C,D,I), compared to negative PAS staining in cerebellum sections from control rats (Fig 4A,B). EPO treatment significantly (p < 0.05) protected against PPA-induced PAS-positive Purkinje cells (Fig 4E,F,I), whereas zinc sulfate provided less protection (Fig 4G-I).

### Correlation between iNOS score and cerebellar injury biomarkers

**Fig 5:**
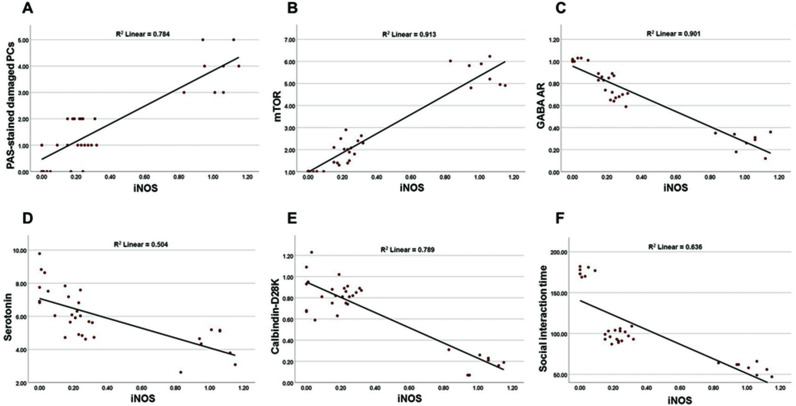
Correlation between the score of Purkinje cells expressing iNOS and cerebellum parameters associated with the induction of autism in rats. All rats had their iNOS expression in Purkinje cells assessed and the relationship between iNOS and glycogen (A), mTOR (B), GABA_A_ receptor (C), serotonin (D), calbindin-D28K (E), and interaction time (F) are shown using Spearman correlation coefficient. iNOS: inducible nitric oxide synthase; PAS: periodic acid Schiff; PC**s:** Purkinje cells; mTOR: mammalian target of rapamycin; GABAa: gamma amino-butyric acid receptor type a; GABA: gamma amino-butyric acid.

The correlation between Purkinje cell iNOS scores deduced from the immunohistochemistry staining data and biomarkers of cerebellar injury (mTOR, PAS-positive Purkinje cells, GABA_A_ receptor, serotonin, calbindin D28K, and social interaction test) was assessed in three groups; control, PPA, and EPO+PPA. This correlation indicates that cerebellar nitrosative stress, limited to damaged Purkinje cells induced by PPA injections, is linked with these parameters associated with cerebellar injury. This finding also supports the beneficial role of EPO in treating serious brain injuries, such as ASD. There was a significant (p < 0.05) correlation between iNOS and PAS-positive Purkinje cells (r =  0.885) (Fig 5A), mTOR (r =  0.956) (Fig 5B), GABA_A_ receptor (r =  - 0.949) (Fig 5C), serotonin (r =  - 0.710) (Fig 5D), calbindin D28K (r =  - 0.888) (Fig 5E), and social interaction measured by the animals’ interaction time (r =  - 0.797) (Fig 5F).

## Discussion

**Fig 6:**
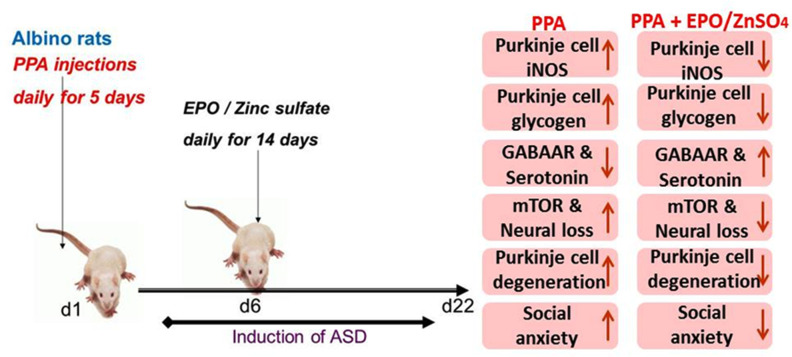
Proposed model for PPA induced autism in rats appears protected by EPO and ZnSO_4_. PPA: propionic acid; EPO: erythropoietin; ZnSO_4_: zinc sulfate; iNOS: inducible nitric oxide synthase; mTOR: mammalian target of rapamycin; GABAa: gamma amino-butyric acid receptor type a; d1: day one; ASD: autism spectrum disorder.

To the best of our knowledge, these studies are the *first* to report an association between Autism Spectrum Disorder (ASD) and co-localization of iNOS and glycogen in degenerated cerebellar Purkinje cells. Additionally, we investigated the differential protection of the cerebellar cortex from PPA-induced ASD by EPO and zinc sulfate, which demonstrated incomparable potency for EPO and zinc sulfate in some of the assessed parameters. Basic and special histological staining methods, physiological, immunohistochemical, biochemical, and molecular approaches were used to test our working hypothesis. These conclusions are supported by data indicating that PPA-induced cerebellar injury in rats (Fig 6) caused an *exclusive* expression of the nitrosative stress marker iNOS, as well as glycogen deposition in damaged Purkinje cells, associated with a sharp decrease in the number of the normal Purkinje cells assessed by basic histology staining. Accumulation of PAS-positive abnormal insoluble glycogen (polyglucosan bodies) in all brain regions of people with the rare autosomal recessive disorder Lafora disease is associated with neuronal loss and myoclonic epilepsy [27,35]. These corroborate our data highlighted in Fig 4 and Fig 3E. However, further work is needed to thoroughly investigate the role of the brain’s *only* stored energy source (glycogen) in ASD.

In addition to the well-known effects of oxidative stress on the development of ASD [24,36], nitrosative stress was also proposed to play a role in the pathophysiology of this disorder and other brain diseases. For example(i) elevated levels of nitric oxide and its metabolites are reported in children with ASD [37]; (ii) an increase in intracellular NO leads to S-nitrosylation of many proteins, which could play a role in the pathology of ASD and Alzhiemer’s disease [38]; (iii) PPA-induced ASD in rats caused a significant increase in iNOS protein levels in mitochondrial complexes isolated by a method that utilized the whole brain excluding the cerebellum, which was inhibited by green tea catechin [39]; (iv) anxiety-like behaviour caused by early maternal separation in rats induced iNOS mRNA in the hippocampus, further increased in the group of rats that received L-arginine, the substrate for nitric oxide synthase (NOS) enzyme, and substantially decreased in rats that received the nonselective NOS inhibitor, L-NAME [25]; (v) a sevenfold increase in iNOS (but not nNOS) concentration in the brain regions of the hypothalamus and thalamus was reported in a rat model of sleeping sickness following infection with the parasite that causes African trypanosomiasis in humans, Trypanosoma brucei brucei [40]. The authors suggested iNOS “as a marker of deleterious inflammatory reactions”; and (vi) a recent report on a model of Alzheimer’s disease induced in rats by aluminium chloride caused the augmentation of cerebellar iNOS protein expression in Purkinje cells, which was inhibited by the flavonoid naringin [41]. These reports corroborate our finding of an association between ASD induced by PPA and immunohistochemistry of iNOS protein in degenerated Purkinje cells, which were significantly protected by EPO and zinc sulfate (Fig 2).

Dysregulation of mTOR is associated with ASD, and targeting mTOR was suggested as a possible therapeutic approach in ASD management [26,42]. For example, treating mice that carry specific gene mutations affecting mTOR activities in either Purkinje cell or the hippocampus with rapamycin, an mTOR inhibitor, prevented ASD pathology and behavioural deficits [43,44]. Rapamycin also inhibited hippocampal long-term depression in mice involving activation of p-mTOR and inhibiting the cognitive deficits associated with mTOR activation in a mouse model of Alzheimer’s disease [45,46]. In another study however, levels of p-mTOR were depressed in the hippocampus, hypothalamus, and prefrontal cortex of allergic mice (induced by cow’s milk allergy) with ASD behavior, increasing only in the amygdala of these animals [47]. Therefore, our novel data pointing to the upregulation (about 80%) of cerebellar mTOR mRNA in rats with ASD, which is demonstrably inhibited by EPO and zinc sulfate (Fig 3), align with previous reports demonstrating an increase in mTOR protein expression activity in various brain regions in animal models of ASD and other neurodegenerative diseases, protected by rapamycin.

Conflicting data on levels of the major inhibitory neurotransmitter receptor in the central nervous system, the GABA_A_ receptor, have been reported in patients and animals with ASD and other neuronal disorders. For example, (i) a significant reduction in GABA_A_ receptor availability was reported in children with ASD [48], and enhancement of GABA_A_ receptor activity in the hippocampus of ASD mice using benzodiazepines reduced autistic-like behaviours [49]; (ii) induction of ASD in rats by a single injection of valproic acid (500 mg/kg) to pregnant rats caused a reduction in GABA_A_ receptor activity in the amygdala in two generations of offspring [50]; (iii) a 63% reduction in GABA_A_ receptor subunit 1 protein in the cerebellum of autistic patients was reported [51]; (iv) activation of GABA_A_ receptor treated sleeping disorder [52]; and (v) brain ischemia reduced GABA_A_ receptor density [53]. These reports align with our data (Fig 3), which show a sharp decline (about 75% reduction) in cerebellar GABA_A_ receptor gene expression in ASD rats, while EPO and zinc sulfate treatments substantially protected the GABA_A_ receptor gene expression levels. However, one study reported no change in any brain region for GABA_A_ receptor availability in ASD patients free of medications and in three ASD mouse models with mutations associated with ASD in humans compared to controls [54].

In summary, we have demonstrated the co-localization of iNOS and glycogen in degenerated Purkinje cells in a rat model of PPA-induced ASD, associated with the upregulation and downregulation of cerebellar mTOR and GABA_A_ receptor, respectively. This provides potential new biomarkers for damaged Purkinje cells in ASD and where the effects of ASD are shown to be ameliorated by EPO and zinc sulfate.

### Limitations of the Study.

A fifth animal group treated with iNOS inhibitor would have been invaluable in the assessment of this novel biomarker, and we also suggest future work using iNOS knockout mice in a mouse model of ASD. Additionally, although currently there is no available drug target to specifically treat ASD, it is worth trying in our rat model of ASD the antipsychotic drug risperidone, the first approved by the FDA to treat the common symptom of irritability in autism, and compare its effectiveness with EPO and zinc sulfate on iNOS as well as other other pre-specified parameters.

## Supporting information

S1 Data ASD supporting raw data. (PDF)
