# Supplementary material for: Identifying iNOS and glycogen as biomarkers for degenerated cerebellar purkinje cells in autism spectrum disorder: Protective effects of erythropoietin and zinc sulfate
Source: PLoS One. 2025 Feb 13;20(2):e0317695. doi: 10.1371/journal.pone.0317695 (PMC11824972; doi:10.1371/journal.pone.0317695)
Supplement: S1 Data — (PDF) [file pone.0317695.s001.pdf]

	Control	Autism	EPO	Zn-
	6	1	3	3
H&E	5	2	4	4
# normal	6	1	4	3
PCs	6	2	4	3
	5	0	5	5
	5	3	5	3
	**6**	**2**	**3**	**5**
	6	2	4	4
**Mean**	**5.625**	**1.625**	**4**	**3.75**
**SD**	**0.5175**	**0.9161**	**0.7559**	**0.8864**

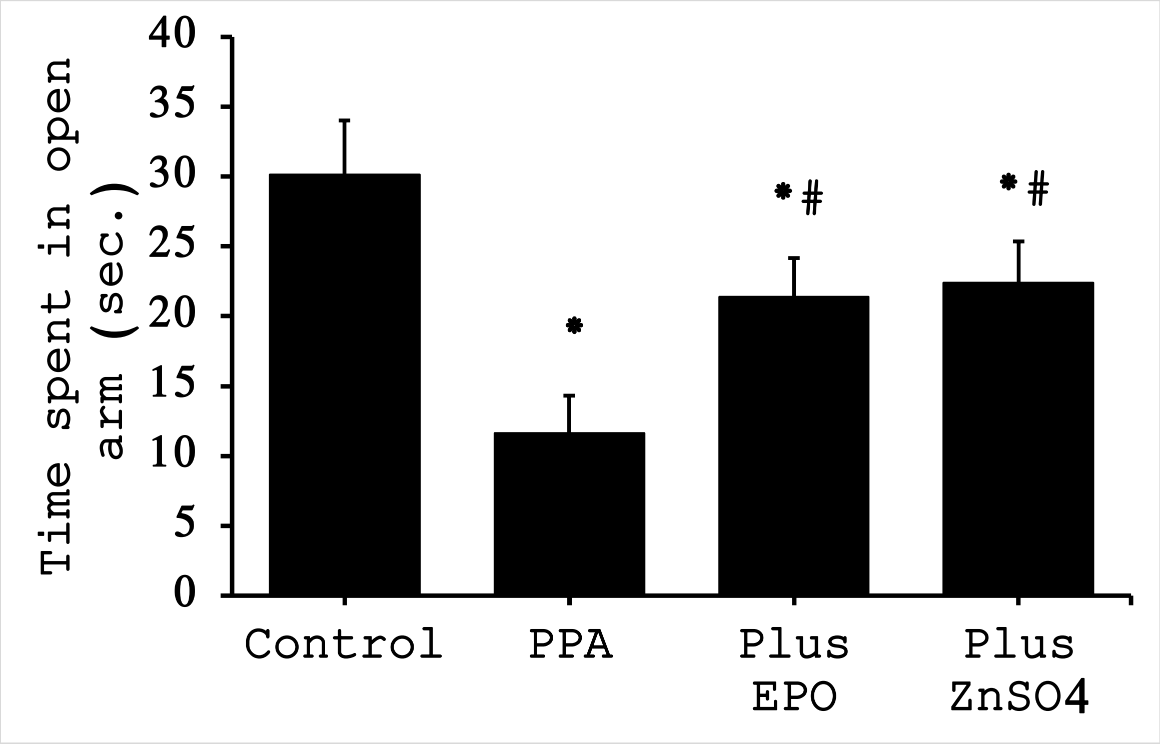

Values are presented as mean ±SD

*: statistically significant compared to corresponding value in control group (P<0.05)

#: statistically significant compared to corresponding value in PPA group (P<0.05)

$: statistically significant compared to corresponding value in EPO+PPA group (P<0.05)

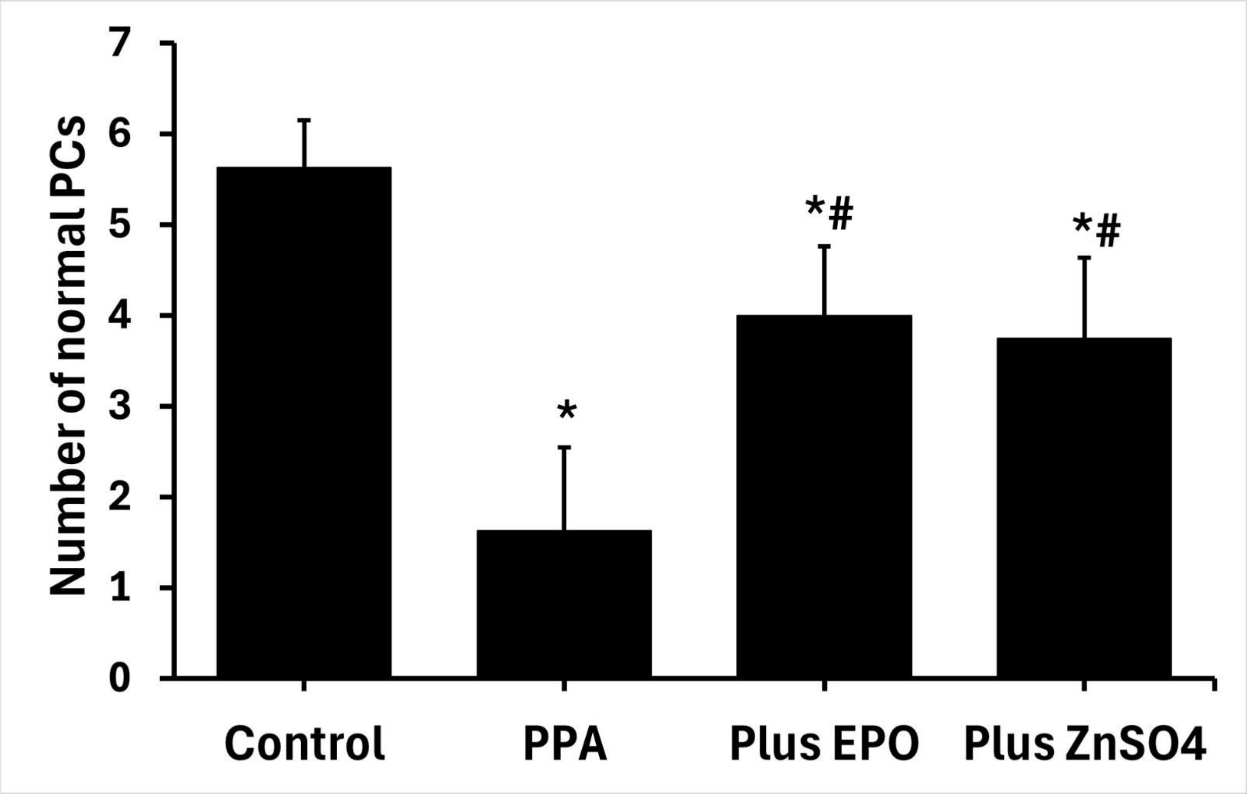

Number of normal PCs
7
6
5
4
3
2
1
0
*
*#
*#
Control
PPA
Plus EPO
Plus ZnSO4

	Control	PPA	EPO+PPA	ZnSO4+PPA
**mTOR**	1.01±0.01	5.48±0.57*	1.71±0.44*#	2.25±0.38*#
**GABA AR**	1.01±0.01	0.28±0.09*	0.85±0.05*#	0.67±0.04*#$

Values are presented as mean ±SD

*: statistically significant compared to corresponding value in control group (P<0.05)

#: statistically significant compared to corresponding value in PPA group (P<0.05)

$: statistically significant compared to corresponding value in EPO+PPA group (P<0.05)

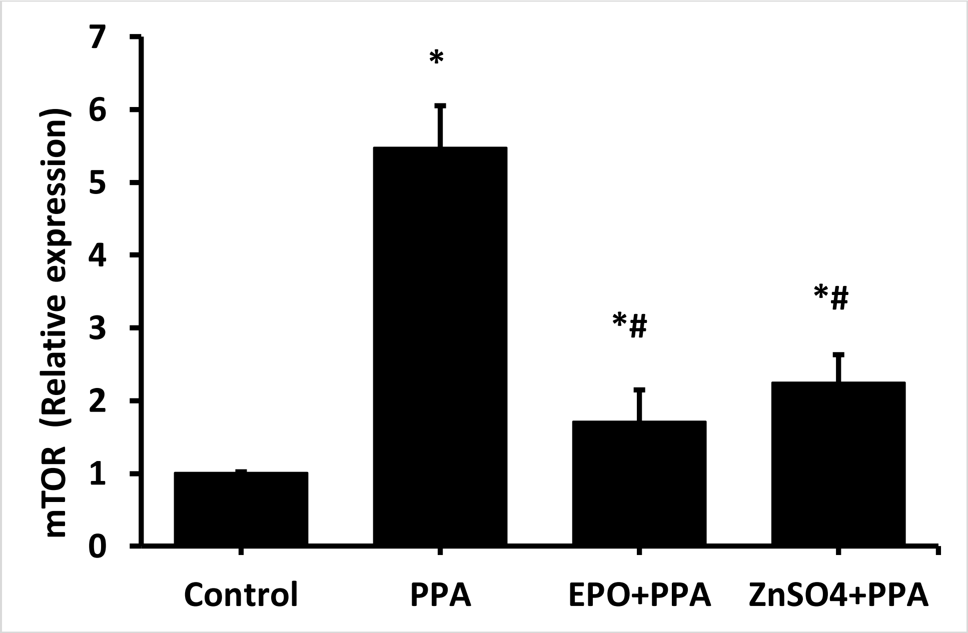

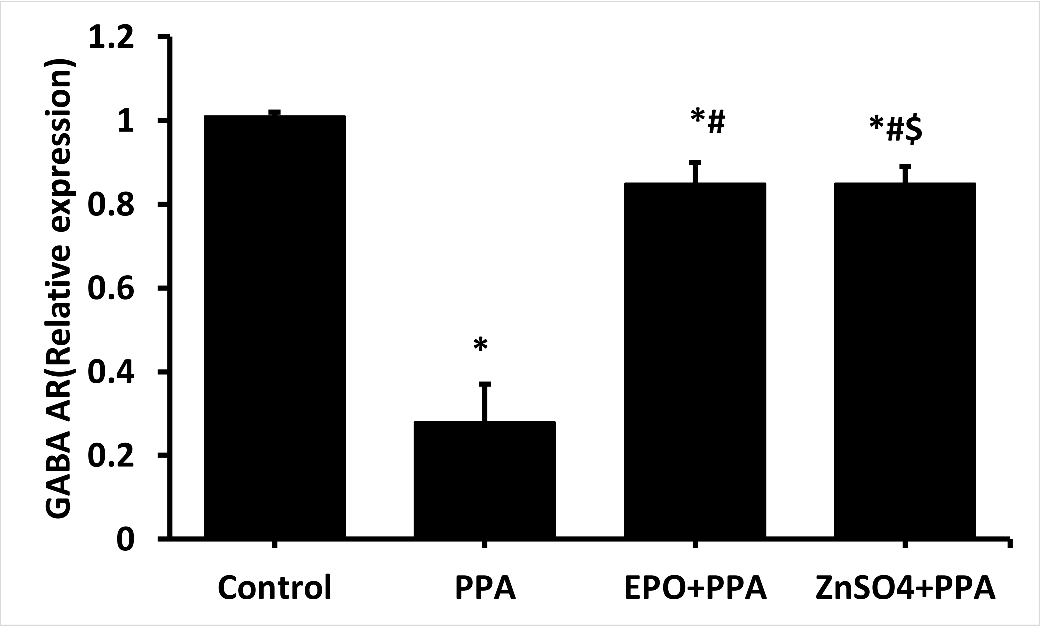
GABA AR(Relative expression)
1.2
1
0.8
0.6
0.4
0.2
0
*
*#
*#$
Control
PPA
EPO+PPA
ZnSO4+PPA

Correlations that include four groups (control, PPA, PPA+EPO, and PPA+ZnSO4)

R square values

	iNOS optical density		
	**Correlation Coefficient**	**P value**	**N**
**No. of open arm entry**	-0.664-	<0.001	32
**Interaction time (sec.)**	-0.839-	<0.001	32
**PAS-stained damaged PCs**	0.759	<0.001	32
**GABA**	-0.666-	<0.001	32
**MTOR**	0.882	<0.001	32
**Serotonin**	-0.795-	<0.001	32
**GABA AR**	-0.917-	<0.001	32
**Calbindin-D28K**	-0.568-	0.001	32

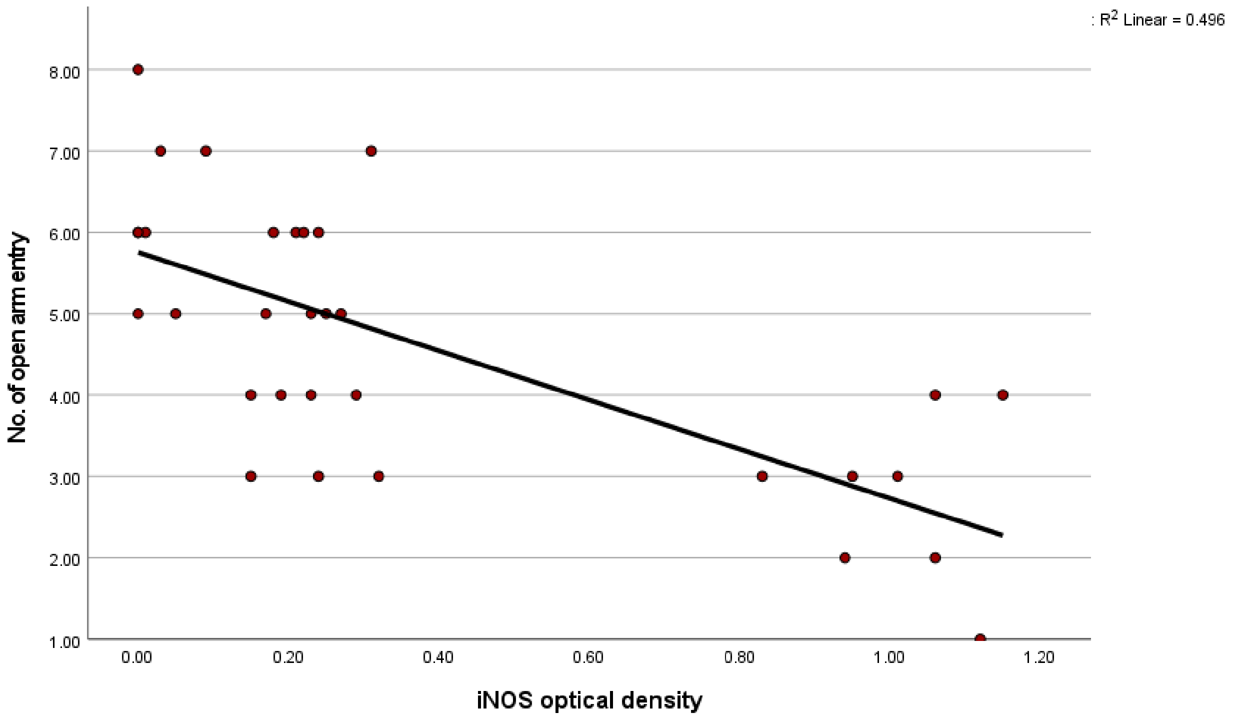

R2 Linear = 0.496
No. of open arm entry
8.00
7.00
6.00
5.00
4.00
3.00
2.00
1.00
0.00
0.20
0.40
0.60
0.80
1.00
1.20
iNOS optical density

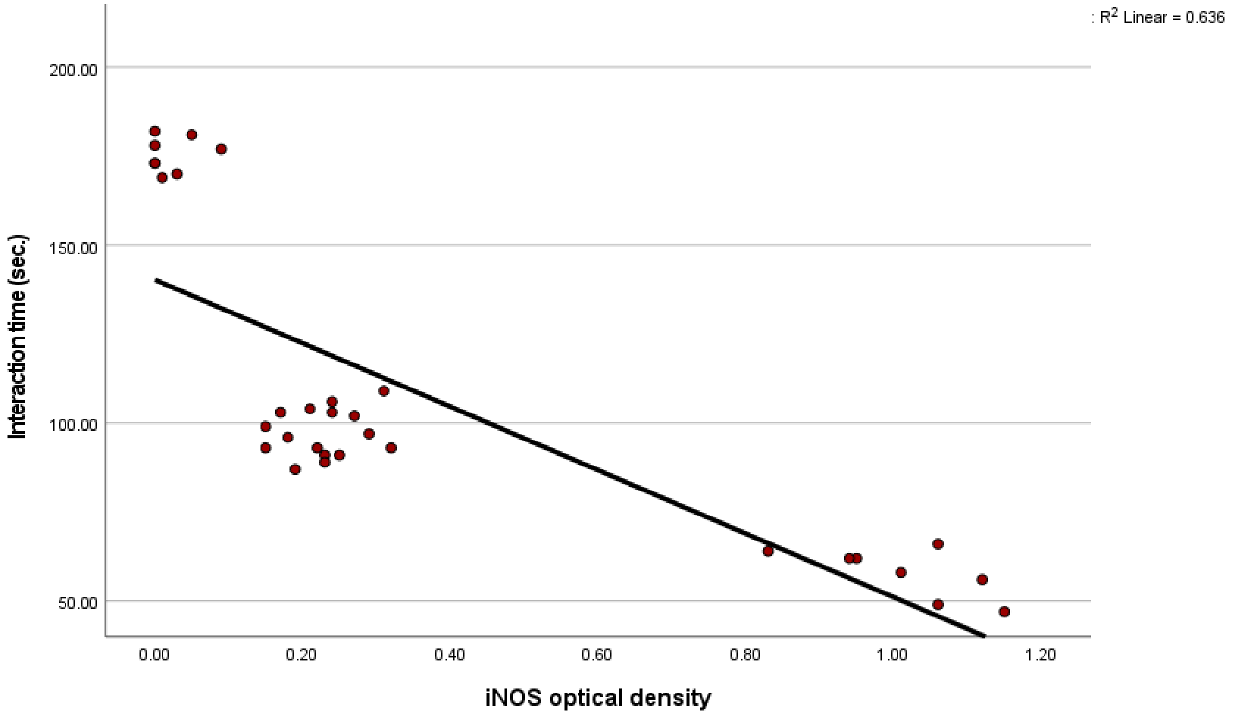

R2 Linear = 0.636
Interaction time (sec.)
200.00
150.00
100.00
50.00
0.00
0.20
0.40
0.60
0.80
1.00
1.20
iNOS optical density

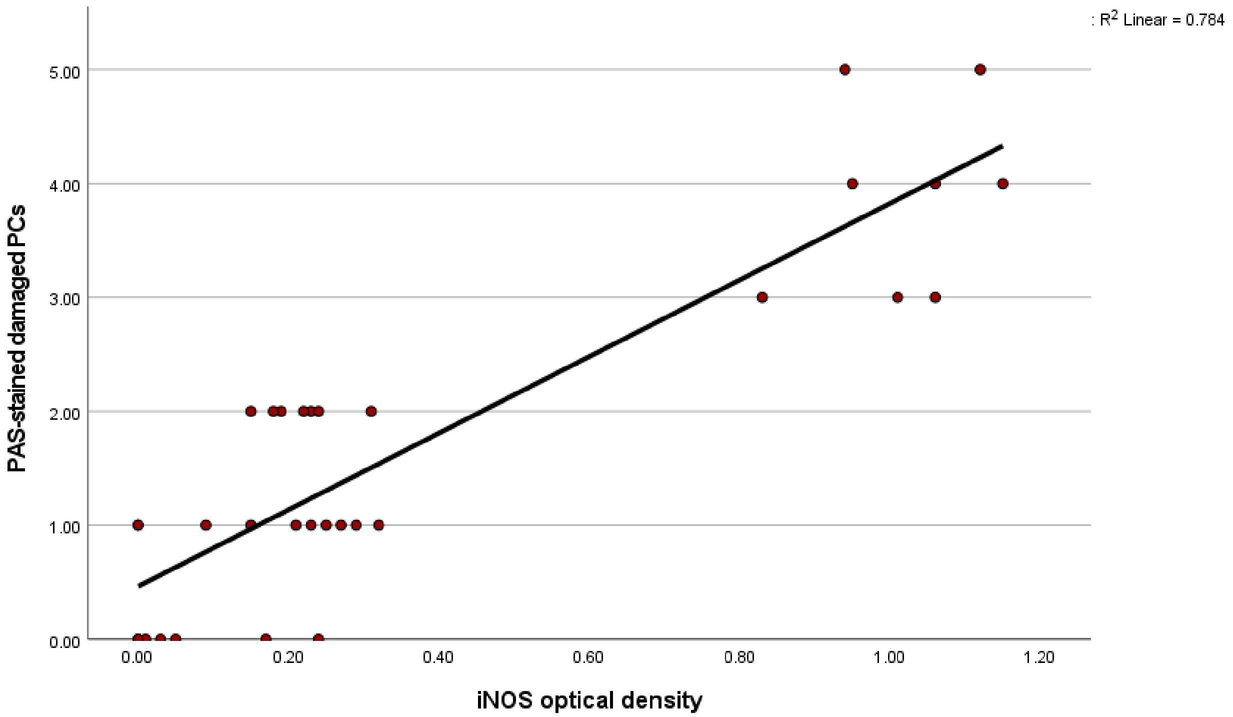
R^2 Linear = 0.784
PAS-stained damaged PCs
5.00
4.00
3.00
2.00
1.00
0.00
0.00
0.20
0.40
0.60
0.80
1.00
1.20
iNOS optical density

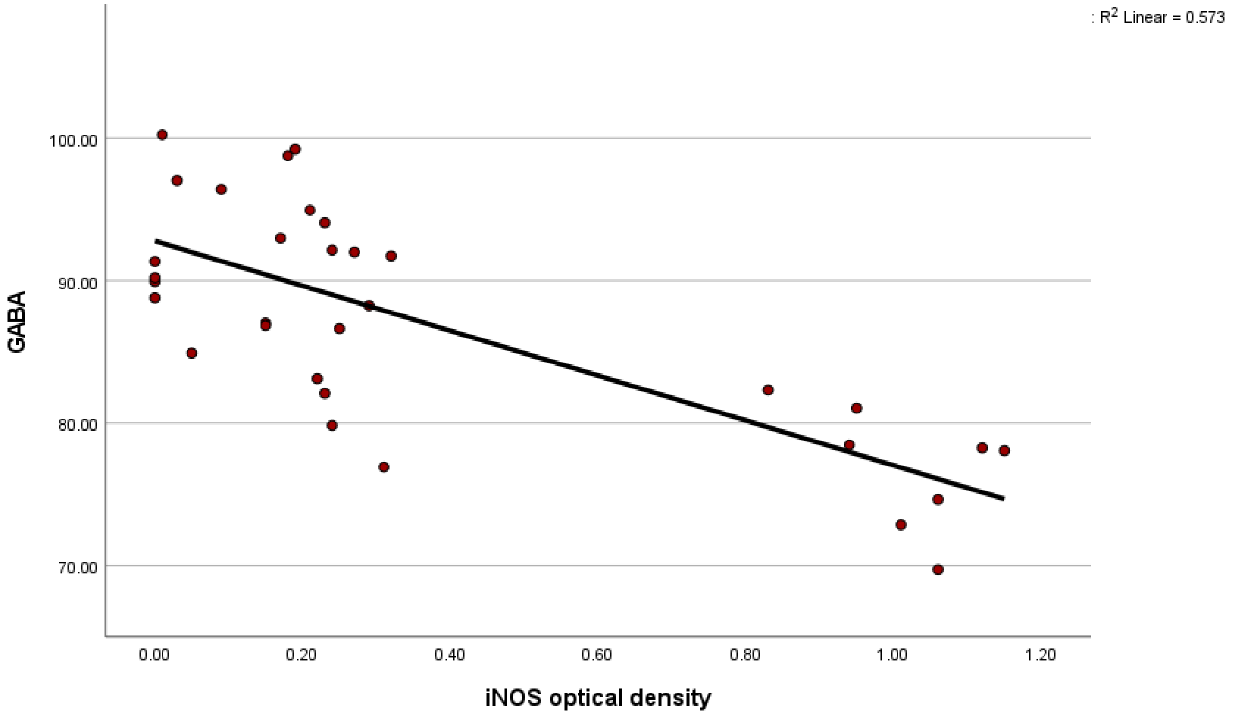
R^2 Linear = 0.573
GABA
100.00
90.00
80.00
70.00
0.00
0.20
0.40
0.60
0.80
1.00
1.20
iNOS optical density

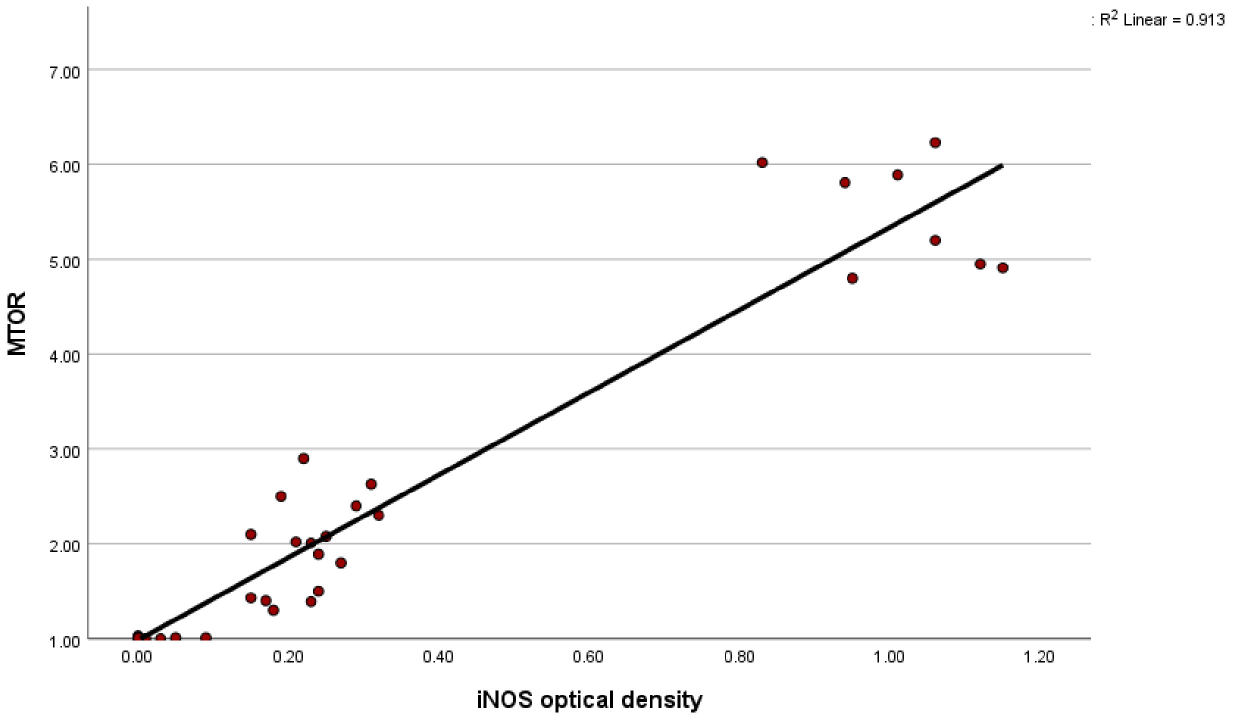
R2 Linear = 0.913
7.00
6.00
5.00
4.00
3.00
2.00
1.00
MTOR
0.00
0.20
0.40
0.60
0.80
1.00
1.20
iNOS optical density

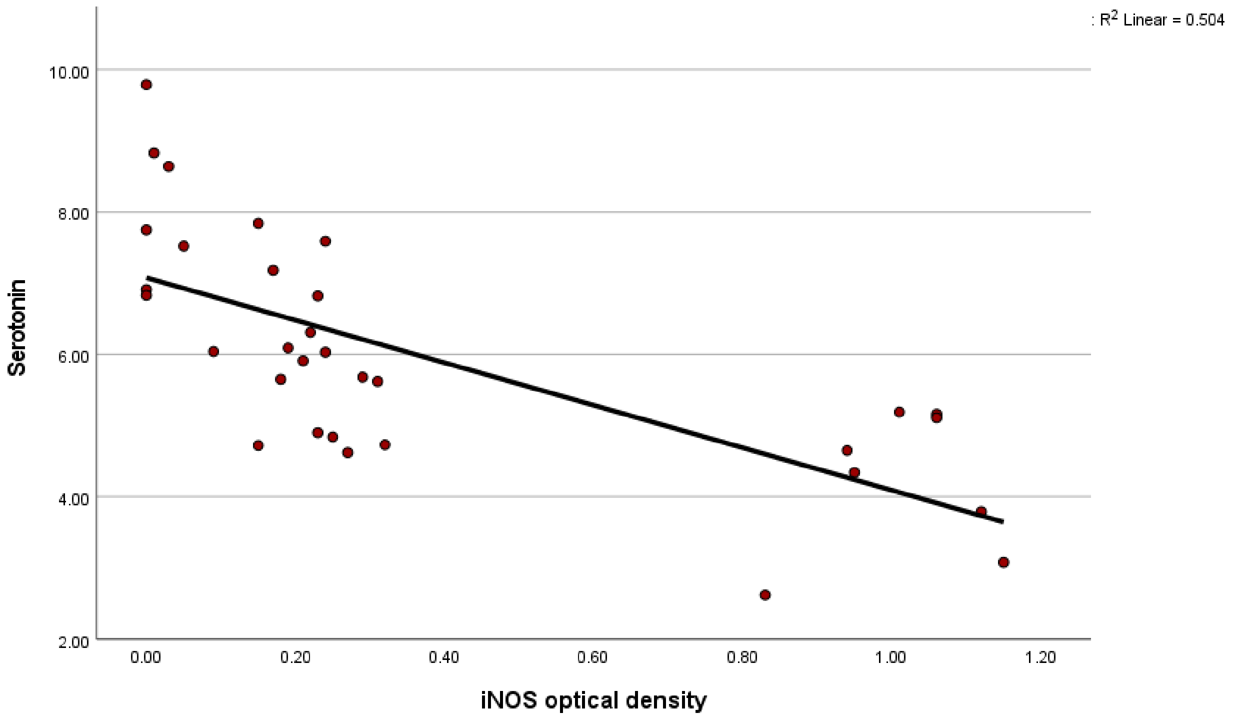
R2 Linear = 0.504
10.00
8.00
6.00
4.00
2.00
Serotonin
0.00
0.20
0.40
0.60
0.80
1.00
1.20
iNOS optical density

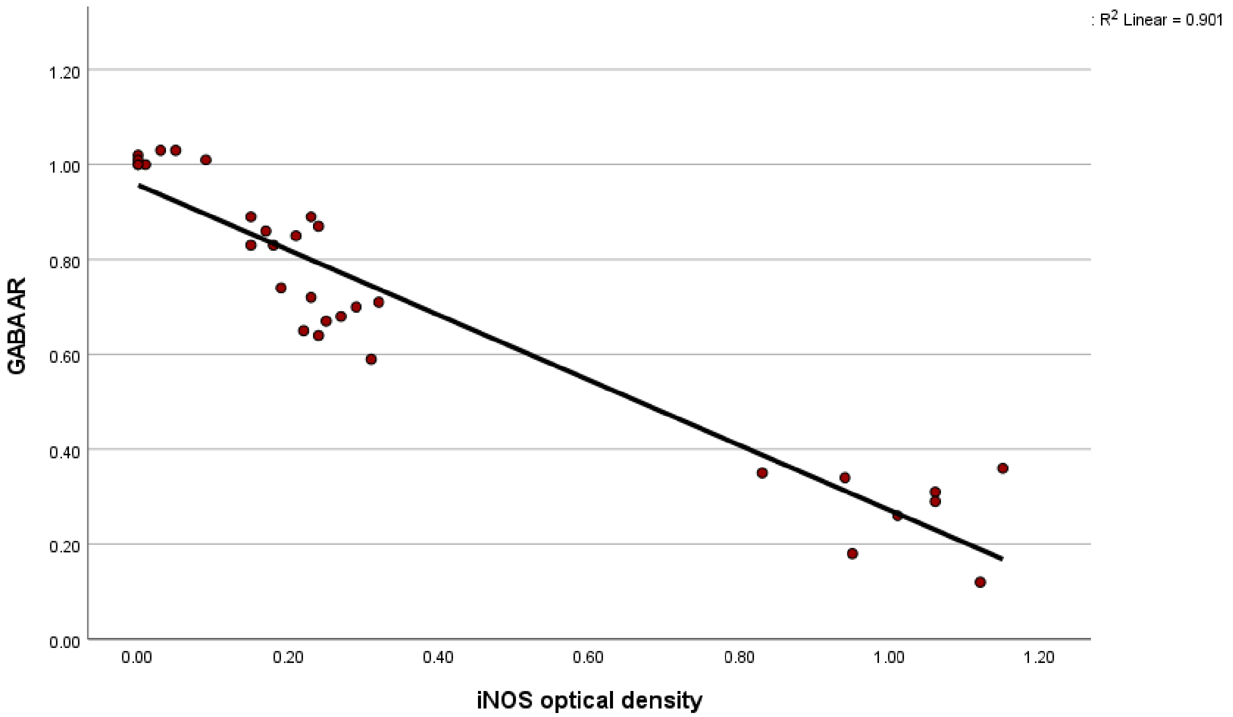

R2 Linear = 0.901
1.20
1.00
0.80
0.60
0.40
0.20
0.00
GABA AR
0.00
0.20
0.40
0.60
0.80
1.00
1.20
iNOS optical density

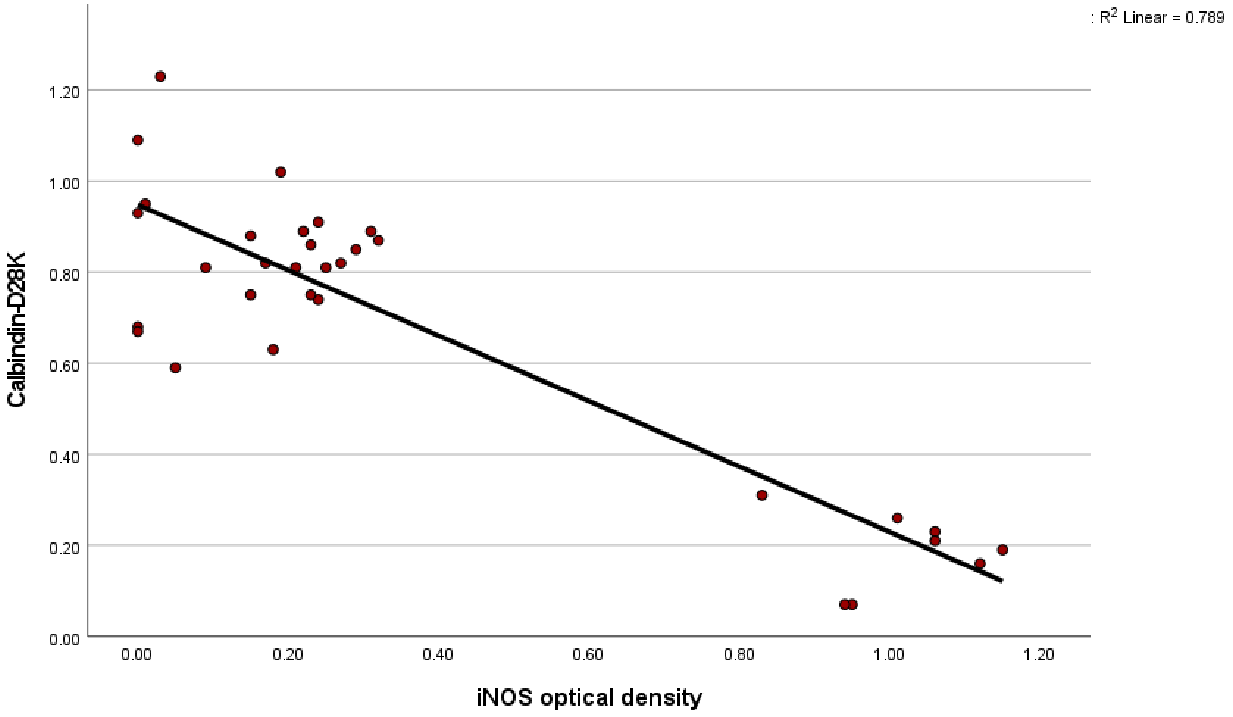

R2 Linear = 0.789
1.20
1.00
0.80
0.60
0.40
0.20
0.00
Calbindin-D28K
0.00
0.20
0.40
0.60
0.80
1.00
1.20
iNOS optical density

2. Two nonparametric graphs for iNOS and PAS

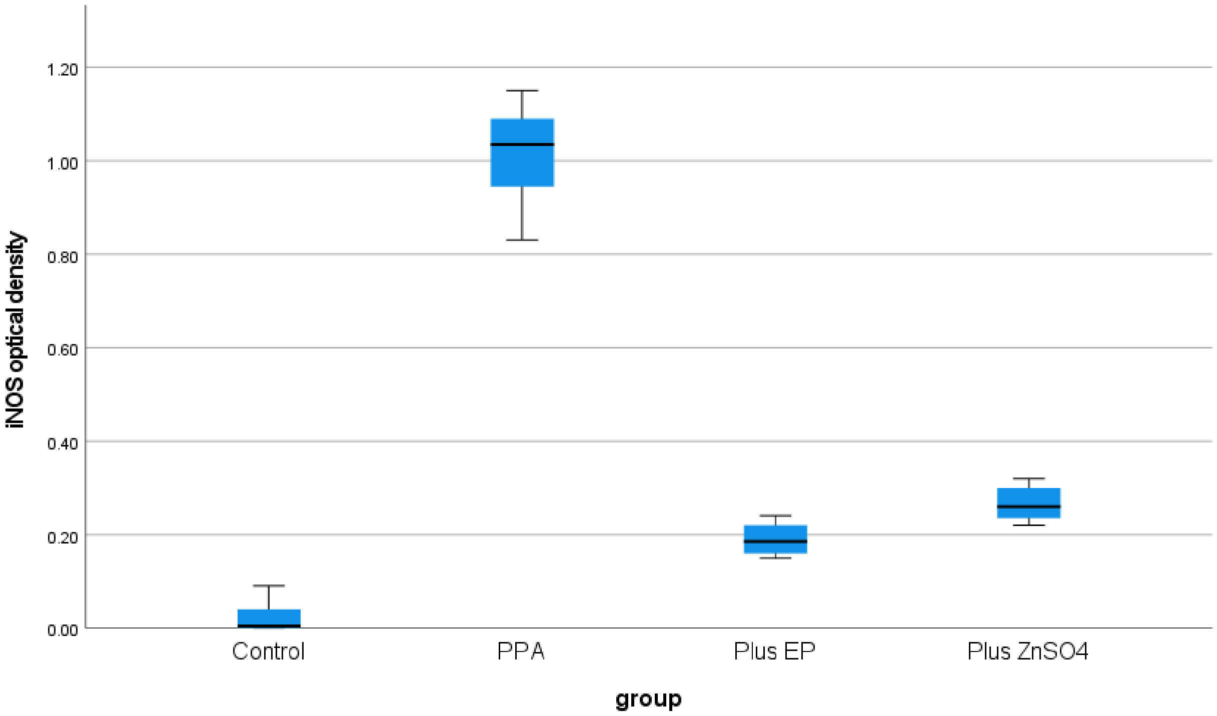

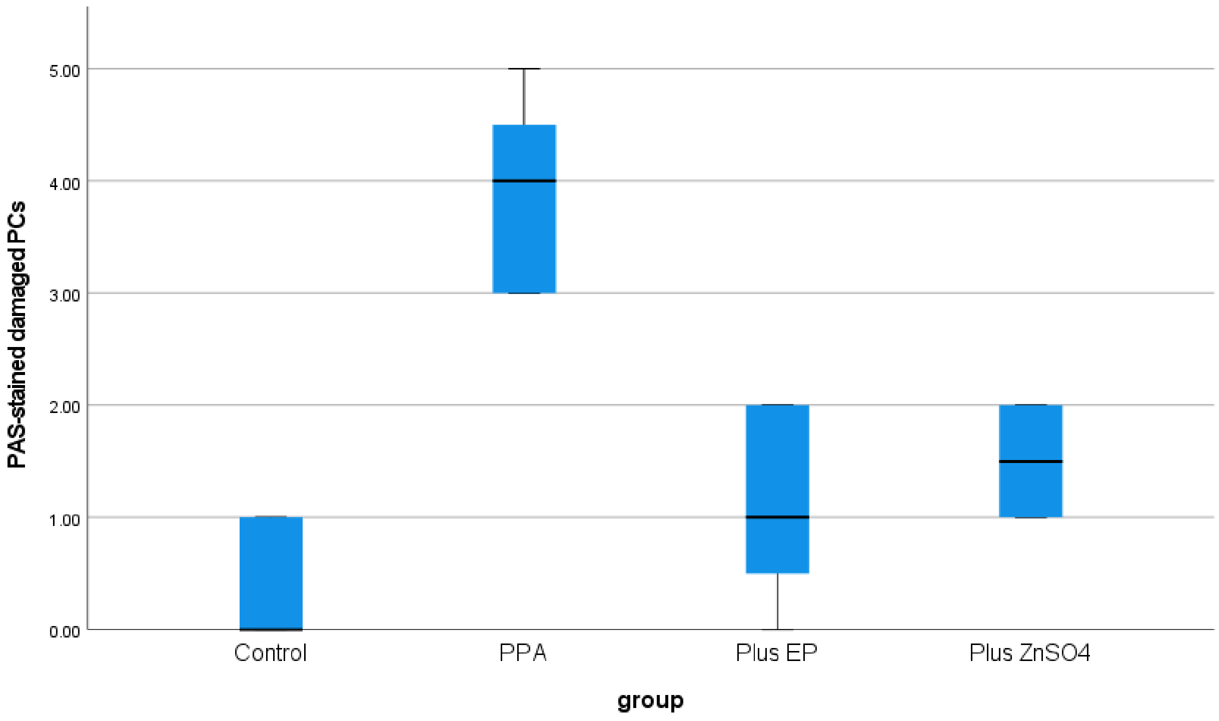

	group												
	Control			PPA			Plus EP			Plus ZnSO4			P value
	Median	1st quartile	3rd quartile	Median	1st quartile	3rd quartile	Median	1st quartile	3rd quartile	Median	1st quartile	3rd quartile	
iNOS optical density	0.01	0.00	0.04	1.04	0.95	1.09	0.19	0.16	0.22	0.26	0.24	0.30	<0.001
PAS-stained damaged PCs	0.00	0.00	1.00	4.00	3.00	4.50	1.00	0.50	2.00	1.50	1.00	2.00	<0.001

P value between each 2 groups

iNOS optical density	P value
Control versus Plus EP	0.048
Control versus Plus ZnSO4	0.006
Control versus PPA	<0.001
Plus EP versus Plus ZnSO4	0.135
Plus EP versus PPA	<0.001
Plus ZnSO4 versus PPA	0.048

P value between each 2 groups

PAS-stained damaged PCs	P value
Control versus Plus EP	0.163
Control versus Plus ZnSO4	0.036
Control versus PPA	<0.001
Plus EP versus Plus ZnSO4	0.485
Plus EP versus PPA	0.001
Plus ZnSO4 versus PPA	0.010
